# Genome-Wide Identification and Expression Analysis of the Plant U-Box Protein Gene Family in *Phyllostachys edulis*

**DOI:** 10.3389/fgene.2021.710113

**Published:** 2021-11-30

**Authors:** Jie Zhou, Yaping Hu, Jiajia Li, Zhaoyan Yu, Qirong Guo

**Affiliations:** ^1^ Co-Innovation Center for Sustainable Forestry in Southern China, Nanjing Forestry University, Nanjing, China; ^2^ International Center of Bamboo and Rattan, Beijing, China

**Keywords:** U-box domain, PUB gene family, *Phyllostachys edulis*, evolution, collinearity, abiotic stresses

## Abstract

The *U-box* gene encodes a ubiquitin ligase that contains a U-box domain. The plant U-box (PUB) protein plays an important role in the plant stress response; however, very few studies have investigated the role of these proteins in Moso bamboo (*Phyllostachys edulis*). Thus, more research on PUB proteins is necessary to understand the mechanisms of stress tolerance in *P. edulis*. In this study, we identified 121 members of the PUB family in *P. edulis* (*PePUB*), using bioinformatics based on the *P. edulis* V2 genome build. The *U-box* genes of *P. edulis* showed an uneven distribution among the chromosomes. Phylogenetic analysis of the *U-box* genes between *P. edulis* and *Arabidopsis thaliana* suggested that these genes can be classified into eight subgroups (Groups I–VIII) based on their structural and phylogenetic features. All *U-box* genes and the structure of their encoded proteins were identified in *P. edulis*. We further investigated the expression pattern of *PePUB* genes in different tissues, including the leaves, panicles, rhizomes, roots, and shoots. The qRT-PCR results showed that expression of three genes, *PePUB15*, *PePUB92,* and *PePUB120*, was upregulated at low temperatures compared to that at 25°C. The expression levels of two *PePUBs*, *PePUB60* and *PePUB120*, were upregulated under drought stress. These results suggest that the *PePUB* genes play an important role in resistance to low temperatures and drought in *P. edulis*. This research provides new insight into the function, diversity, and characterization of *PUB* genes in *P. edulis* and provides a basis for understanding their biological roles and molecular mechanisms.

## Introduction

The ubiquitin-proteasome pathway (UPP) is the most important and best characterized pathway for selective protein degradation in eukaryotic organisms. It is also an important mechanism for the regulation of cellular functions such as *Arabidopsis thaliana* (Vierstra, 2009). Previous studies have shown that the UPP is closely connected to plant signaling, development, biotic and abiotic stresses, and ubiquitination (Zhang and X., 2005; Stone et al., 2006; Zhang et al., 2007). Ubiquitin (Ub) is a 76 amino acid (aa) protein that is widely found in eukaryotic organisms, and is highly conserved in sequence (Azevedo et al., 2001). The sequence of Ub amino acids in various higher plants analyzed thus far is almost identical, differing from the human ubiquitin sequence by only three amino acids (Callis and Vierstra, 1989). UPP ubiquitination of the substrate is completed by the cascade reaction of three key enzymes: Ub-activating enzyme (E1), ubiquitin-conjugating enzyme (E2), and ubiquitin-protein ligase (E3). In this pathway, E3 is the most abundant enzyme in the ubiquitin connection cascade system (Parveen et al., 2021). The *Arabidopsis thaliana* genome is estimated to contain more than 1,300 genes encoding E3s, making up more than 5% of the *A. thaliana* proteome, and these are responsible for controlling the regulation of nearly 2,600 target proteins (Zeng et al., 2008; Schulman and Wade Harper, 2009; Ye and Rape, 2009). Each cascade reaction pathway can be used as a targeted method to identify specific degradation signals. E3s in plants can be divided into four types: Homology to the E6AP C-Terminus (HECT) (Metzger et al., 2012), real interesting new gene (*Ring*)/U-box (Cyr et al., 2002), Skp1-cullin-F-box (SCF) (Gagne et al., 2002), and the anaphase-promoting complex (Harper et al., 2002). The U-box type E3 ubiquitin ligase contains a U-box domain consisting of 75 aa, which is a variant of the RING-type ring finger structural domain. The main difference between the U-box type and the RING-type structural domains is that the U-box domain maintains its conformation throughout interactions with components such as hydrogen and salt bonds (Donna and Daphne, 2009; Dielen et al., 2010; Metzger et al., 2012). Analysis of their structure provides an in-depth understanding of the mechanism of action of these enzymes (Hellmann, 2002; Vierstra, 2009; Downes et al., 2010).

*PUB* genes are widely expressed in plants and have been reported in many higher plants, including *A. thaliana* (Jakob et al., 2008), rice (Zeng et al., 2008), cotton (Lu et al., 2020), soybean (Ning et al., 2016), Chinese cabbage (Wang et al., 2015), banana (Hu et al., 2018), and apples (Wang et al., 2020). Currently, 64 *PUB* genes, which perform multiple functions, have been identified in *A. thaliana* (Jakob et al., 2008). There are 125 *PUB* genes in soybeans, which are divided into eight subtypes based on their protein domains (Ning et al., 2016). A total of 101 *PUB* genes have been identified in Chinese cabbage, which can be divided into 10 subtypes according to their protein domains (Wang et al., 2015). In rice, 77 *PUB* genes have been identified, which can be divided into nine subtypes. Several studies have shown that PUB proteins are involved in the regulation of plant hormone signal transduction, stress tolerance, and insect resistance (Zeng et al., 2008). In the pepper plant, *CaPUB1* acts as an E3 ubiquitin ligase to negatively regulate dehydration and high salt stress. *CaPUB1* achieves this by promoting the degradation of the 26 S proteasome subunit through the ubiquitination pathway (Cho et al., 2008). Overexpression of *CaPUB1* in transgenic tissues significantly upregulates the expression of the drought stress marker gene, *RD29a* (Cho et al., 2006). *CaPUB1* homologs in *A. thaliana*, *AtPUB22* and *AtPUB23*, can also interact with the 26 S proteasome subunit *AtPRN12a*. *PUB22* and *PUB23* double mutants display increased resistance to drought and salt stress, with *AtPUB22* and *AtPUB23* showing functional redundancy (Cho et al., 2008). Moreover, the *A. thaliana PUB22/23/24* genes belonging to the Group II subtype, have been shown to play a role in plant resistance. The *PUB22/23/24* triple mutant displays an enhancement in the pattern-triggered immunity (PTI) pathway activated by pathogen-associated molecular patterns that results in improved resistance to bacteria (Trujillo et al., 2008; Chen et al., 2014). Furthermore, *AtPUB30* in *A. thaliana* is induced by salt stress, with *AtPUB30* mutant tissues being more resistant to salt stress during seed germination (Hwang et al., 2015).

Mosa bamboo (*Phyllostachys edulis*) is a uniaxial scattered bamboo species belonging to the Gramineae family. *P. edulis* is native to China and is one of the most important bamboo species in the world, occupying the largest cultivation area and possessing the greatest economic value (Fu, 2001; Vorontsova et al., 2016). *P. edulis* was introduced to Japan in 1736 and has since become the second most economically valuable bamboo species in the country. *P. edulis* was later introduced to South Korea, the Philippines, Myanmar, Vietnam, Thailand, India, Europe, and America (Fu, 2001). This species is characterized by rapid growth, which is affected by various environmental factors (Banik, 2015). Further research on its growth and resistance to environmental stresses is important to improve cultivation and downstream applications. Sequencing of the *P. edulis* V2 genome was completed in 2018 (Zhao et al., 2018), and multiple growth (Wang et al., 2019; Yang et al., 2019; Lan et al., 2020; Wang et al., 2021) and stress-related genes (Hou et al., 2018; Cheng et al., 2019; Hou et al., 2020; Liu et al., 2020) have been extensively studied. However, research on the *PUB* gene family in *P. edulis* is lacking. In this study, we used the *P. edulis* V2 genome build (Zhao et al., 2018) to conduct a genome-wide identification of *PUB* genes and analyze of their gene structure and evolution. We also investigated the expression of *PUBs* in different tissues. This research provides a reference for future follow-up and in-depth studies of *PUB* gene function in *P. edulis*.

## Materials and Methods

### PUB Gene Identification

The hidden Markov model (HMM) file corresponding to the U-box domain (PF04564) was obtained from the Pfam database (http://pfam.xfam.org/) (Mistry et al., 2021). HMMER 3.0 was used to search the U-box domain from the Genome Database of Bamboos (http://www.bamboogdb.org/#/) (Zhao et al., 2014). To ensure accuracy, BLAST 2.9.0+ (http://www.bamboogdb.org/#/blast) (Priyann et al., 2019) was used for BLASTP (e > 1.0 e-10) screening of homologous genes from previously identified *P. edulis PUB* genes. The PFAM (Mistry et al., 2021) and SMART (Letunic et al., 2021) programs were used to verify the presence of the U-box core sequence. Candidate genes that potentially contain the U-box domain based on the HMMER results were further individually verified by manual inspection. The tools on the ExPasy website (Panu et al., 2012) were used to obtain the sequence length, molecular weight (MW), and isoelectric point (pI). The subcellular localization of PePUB proteins was predicted using the online tool Plant-mPLoc (http://www.csbio.sjtu.edu.cn/bioinf/plant-multi/) (Chou and Shen, 2010).

### Phylogenetic Analysis and Classification of Moso Bamboo PUB Gene Family

A phylogenetic tree for the sequence alignment of *PePUB* genes was constructed using the maximum parsimony (MP) method in MEGA 7.0. The bootstrap replications were set to 1,000 to estimate the accuracy of the phylogenetic tree. EvolView (Subramanian et al., 2019) was used to visualize the unrooted phylogenetic tree. The PUB protein sequence of *P. edulis* was analyzed using the online program MEME (https://meme-suite.org/meme/tools/meme) (Bailey and Elkan, 1994) with parameters set to maximum motifs of 10, and the best group width set to between 6 and 50.

### Chromosomal Distribution, Gene Duplication and Synteny Analysis

The BLAST module in TBtools software (Chen et al., 2020) was used for pairwise comparisons of the genomes of *P. edulis* against *Oryza sativa* (Ouyang et al., 2007) and *P. edulis* against *A. thaliana* (Cheng et al., 2017) The Multiple Collinearity Scan toolkit (MCScanX) (Wang et al., 2012), with default parameters, was used to obtain the chromosome distribution and interspecific covariance of the PUB gene family. The intraspecific and interspecific covariance results were visualized using Circos (http://circos.ca/) (Krzywinski et al., 2009) and multiple synteny plots (TBtools module) (Chen et al., 2020).

### Cis-Regulatory Elements of PUB Genes in *Phyllostachys edulis*

The 2,000 bp sequence upstream of *PePUB* genes was extracted as the promoter sequence and used in the PlantCare tool (http://bioinformatics.psb.ugent.be/webtools/plantcare/html/) (Lescot et al., 2002) to identify cis-acting elements.

### Plant Materials and Growth Conditions

The seeds of wild-type *P. edulis* were obtained from Guilin, Guangxi Province (110.38, 25.51) and planted in the White Horse Nursery of Nanjing Forestry University (119.18, 31.61). The seedlings were transplanted into an incubator, and the cycle conditions were 24°C with light for 16 h and 21°C without light for 8 h. Three-year-old *P. edulis* seedlings with an above ground height of approximately 80 cm were treated with 20% PEG 6000 and a temperature of 4°C to simulate drought and cold stress, respectively. Young leaves in a good growth state were collected at 0, 3, 6, and 12 h of treatment, snap frozen in liquid nitrogen, and stored at -80°C until RNA extraction.

### Tissue Expression Patterns of *PePUB* Genes

To study the tissue expression patterns of the *PePUB* genes, transcriptome data from different tissues were obtained through the NCBI Short Read Archive database (https://www.ncbi.nlm.nih.gov/sra/) (accession number SRX2408,703) (Peng et al., 2013). The method of analysis was previously described by Peng et al., 2013.

Expression abundance is described as transcripts per million (TPM). TBtools Amazing Heatmap (Chen et al., 2020) was used to draw gene expression heat maps using a log2 (TPM + 1) scale.

### Total RNA Extraction and cDNA Synthesis

Total RNA was extracted using the TRIzol reagent kit (Invitrogen Scientific, Inc., Carlsbad, CA, United States) according to the manufacturer’s protocol. RNA quality was assessed using an Agilent 2,100 Bioanalyzer (Agilent Technologies, Palo Alto, CA, United States) and RNase-free agarose gel electrophoresis. After total RNA was extracted, eukaryotic mRNA was enriched using Oligo (dT) beads, while prokaryotic mRNA was enriched by removing rRNA using the Ribo-Zero^TM^ Magnetic Kit (Epicenter, Madison, WI, United States). The enriched mRNA was then fragmented using fragmentation buffer, and reverse transcribed into cDNA using random primers. Second-strand cDNA was synthesized using DNA polymerase I, RNase H, and dNTPs. cDNA was stored at −20°C for until use.

### Quantitative Real-Time PCR Analyses

Primer Express 5.0 (Singh and Pandey, 2015) was used to design gene-specific primers (listed in Schedule 1). Primer specificity was confirmed by a BLAST search using data downloaded from the BambooGDB and local CDS databases (http://www.bamboogdb.org/#/blast). The tonoplast intrinsic protein (TIP41) (Supplementary Table S6) was used as the reference gene (Fan et al., 2013). qRT-PCR was performed using SYBR Green Master Mix (Applied Biosystems) and samples were run in a CFX 96 Real-Time system (Applied Biosystems). The cycle conditions were as follows: denaturation for 5 s at 95°C, annealing at 55°C for 15 s, and extension at 65°C for 5 s. To ensure the accuracy of the results, three biological replicates and three technical replicates were performed for each sample. PCR products from each sample were verified by DNA sequencing. The fold change in RNA transcripts was calculated using the 2^−ΔΔCt^ method.

## Results

### Identification of the PePUB Proteins in Moso Bamboo

HMMER 3.0 analysis identified 120 candidate genes corresponding to the Pfam PUB family, while BLASTp identified 100 candidate genes. *P. edulis* transcriptome data were also used to confirm candidate genes. Of the 220 identified candidate genes, 16 were found to have been incorrectly identified and 83 were redundant. The remaining 121 candidate genes were annotated as *PePUB* genes, based on the presence of a complete U-box domain. These genes were renamed *PePUB01* to *PePUB121* based on their chromosomal location (Supplementary Table S1).

We analyzed the gene characteristics, including the Gene ID, chromosomal location, length of the open reading frame, length of the protein sequence, protein MW, theoretical pI, and subcellular localization (Supplementary Table S1). Among the 121 *PePUB* genes, *PePUB118* was identified as the smallest protein (188 aa), while *PePUB117* was the largest (1,359 aa). The MW of the proteins ranged from 8.21 to 117.54 kDa, and the pI ranged from 4.65 (*PePUB92*) to 9.49 (*PePUB51*). The subcellular localization of all 121 *PePUBs* was predicted to be in the nuclear region, while six *PePUBs* were also located in the chloroplast.

Figure 3A shows the chromosomal location of the *PePUB* genes. Of the 121 *PePUB* genes, 117 could be mapped to the 20 chromosomes that make up the genome of *P. edulis*. However, four genes (PH02Gene24079.t1, PH02Gene49698.t1, PH02Gene50722.t1, and PH02Gene51283.t1) could not be localized to specific chromosomal locations using the *P. edulis* V2 genome. The largest number of *PUB* genes was found on chromosome 17, which contained 18 *PePUB* genes. Chromosomes 8 and 13 contained 11 *PePUB* genes. Nine *PePUB* genes were found on chromosome 3. Chromosomes 6, 23, and 24 each contained eight *PePUB* genes. Six *PePUB* genes were located on chromosome 20. Chromosomes 5 and 16 each contained four *PePUB* genes. Chromosomes 12 and 22 each contained three *PePUB* genes. Chromosomes 7, 9, 11, and 18 each contained two *PePUB* genes. Only one *PePUB* gene was located on chromosome 19.

### Multiple Sequence Alignment, Phylogenetic Analysis, and Classification of *PePUB* Genes

**FIGURE 1:**
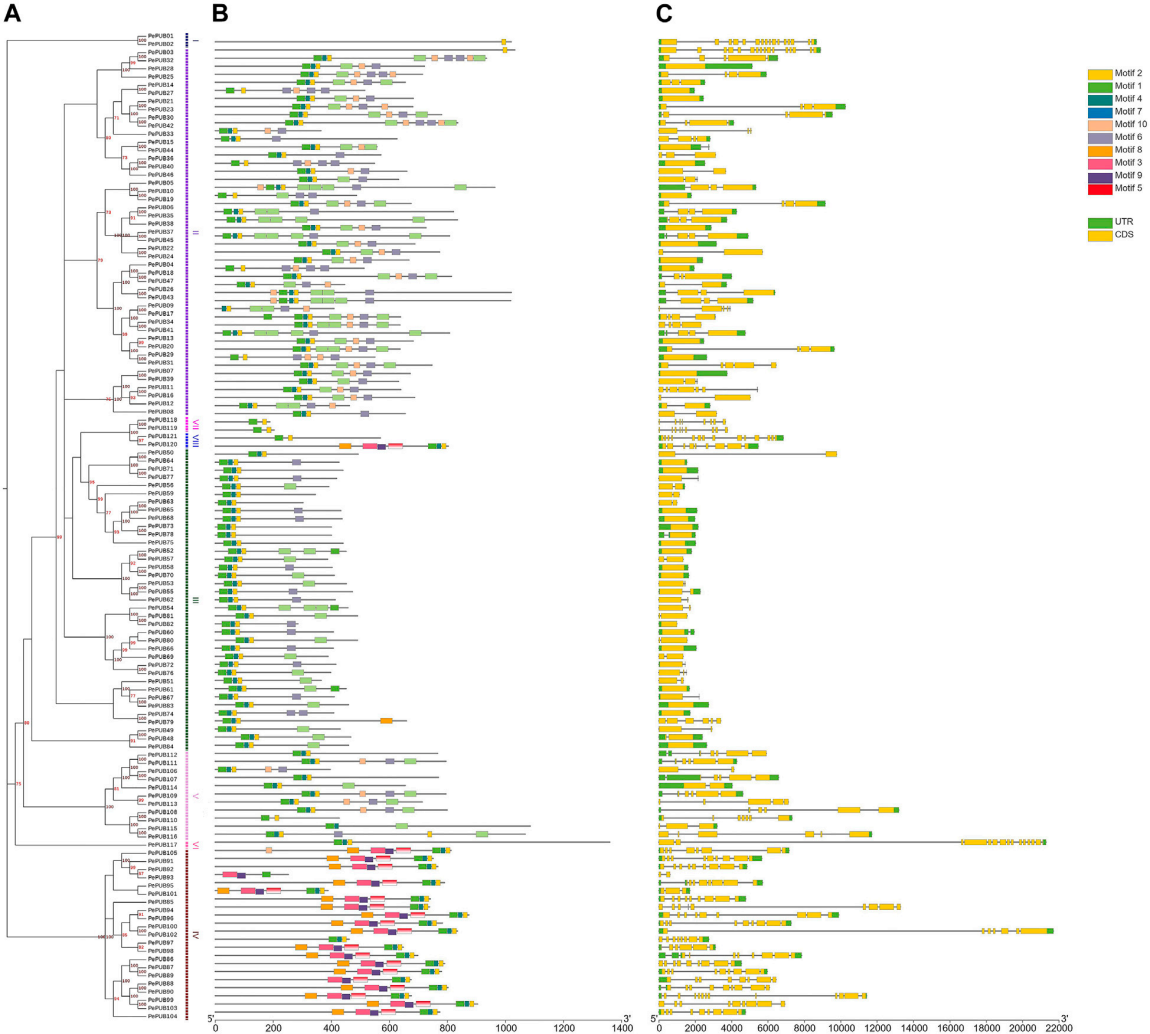
The unrooted phylogenetic tree, motif analyses, and the gene structure of *PePUB* genes. **(A)** The neighboring tree on the left consists of 121 *PePUB* proteins from *Phyllostachys edulis*. The right side of the phylogenetic tree lists the groups. **(B)** The 10 conserved groups are indicated by different colored boxes. **(C)** Yellow indicates the untranslated 5 and 3′ regions, and green indicates the exons.

A phylogenetic tree was constructed using the neighbor-joining method to investigate the relationships among the 121 *PePUB* genes. Phylogenetic analysis showed that these genes can be classified into eight subtypes (Groups I-VIII; Figure 1A). The protein sequence of all 121 *PePUB* genes are listed in Supplementary Table S1. Of the eight subgroups, Group II is the largest with 45 genes. Group VI was the smallest with only one gene.

To gain a more complete understanding of the composition and function of the *PePUB* genes, we investigated conserved sequences and exon/intron positions. Gene structure analysis indicated that the number of exons in *PePUB* genes varied from 0 to 15. Phylogenetic results based on the *PUB* gene family indicated that members of the same subgroup had similar exon/intron structures (Figure 1C). Most of the 37 genes in Group III contained one to three exons, while genes in Group I contained the most exons with all the genes in this group having 16 exons. In conclusion, the coding region structure of the *PePUB* genes are diverse, but there is some similarity among members of the same subgroup. This gene structure analysis corroborated the results of our phylogenetic analysis.

To further investigate the different structures of *PUB* genes, specific base sequences were evaluated using the MEME program. Ten conserved base sequences were identified and predicted for the 121 *PePUB* genes. The results showed that genes in the same subgroup exhibited a conserved base sequence with similar functions. At least one to four primary base sequences were present in all amino acid sequences (1, 2, 4, and 7; Figure 1B). Base sequences one and four are conserved U-box sequences, whereas the other sequences remained unpredictable.

**FIGURE 2:**
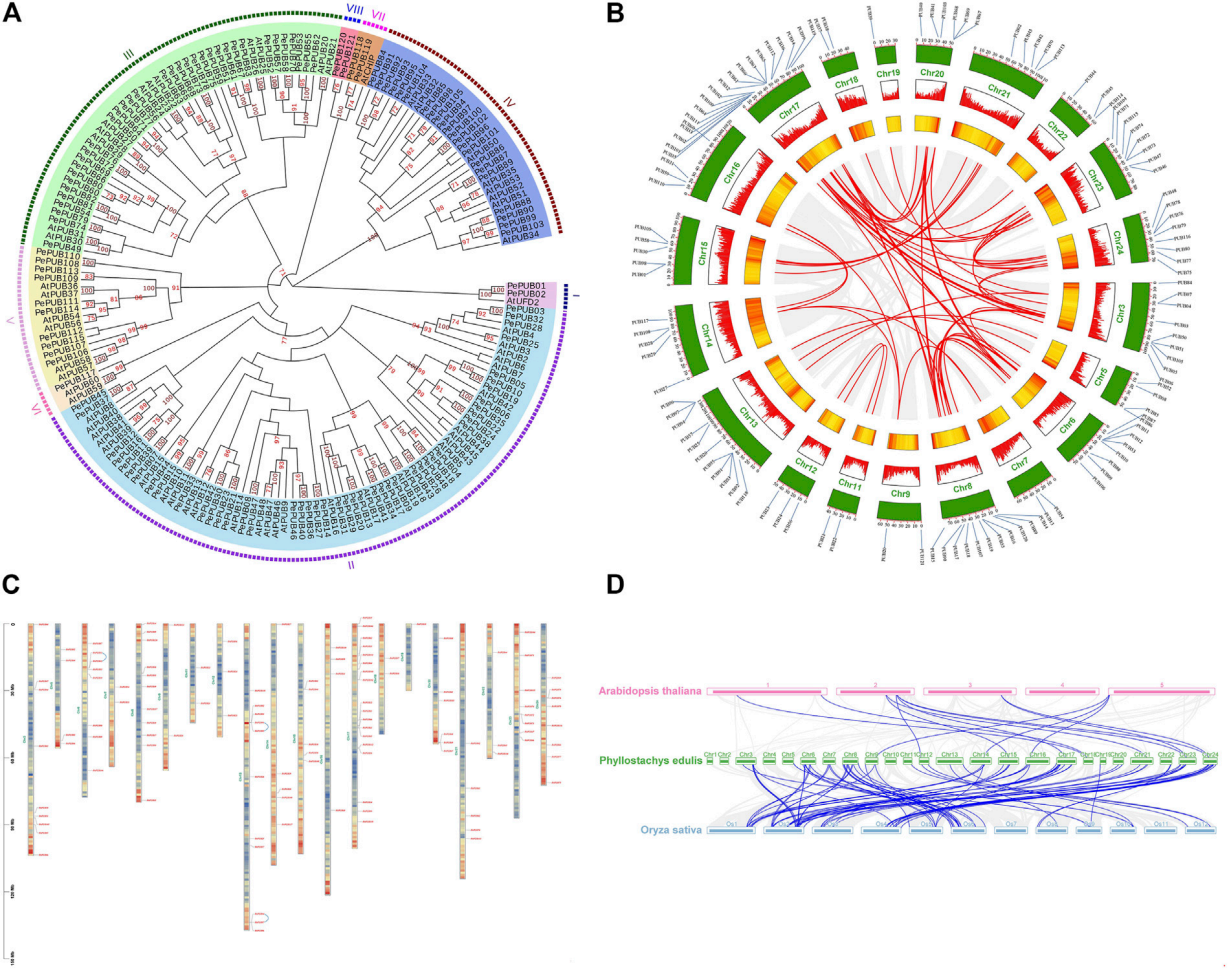
Localization and synteny of *PePUB* in *Phyllostachys edulis* genome. **(A)** Phylogenetic analysis of *PUB* genes from *Phyllostachys edulis* and *Arabidopsis thaliana*. **(B)** Localization of *PUB* on the chromosomes. Chromosome or scaffold number was indicated on the outer side. Yellow represented different chromosomes. The scale in the figure represents the chromosome length (Mb). The different colors represent the gene density of the chromosomes (increasing gene density from blue to red). The ligature between genes represents the tandem duplication event. The Whole genome duplications (WGD)/segmental duplication genes were connected by blue lines. **(C)** Distribution of gene duplications among *PePUB* genes. **(D)** Collinearity analysis between *Phyllostachys edulis* and *Arabidopsis thaliana* and *Oryza sativa*. The gray line represents the co-collinearity of all genes among the three species, and the blue line represents the collinearity among members of the *PUB* gene family.

To further investigate the evolution of the PUB family, 182 PUB proteins (121 from *P. edulis* and 61 from *Arabidopsis thaliana*) (Supplementary Table S2) were used to generate a phylogenetic tree (Figure 2A). We found that the PUB protein groupings were consistent with previous results and could be divided into eight subgroups. This further supports the accuracy of our results.

### Chromosomal Distribution and Gene Duplication of Moso Bamboo *PUB* Gene Family

The chromosome distribution of the *PePUB* gene family is shown in Figure 2B. A total of 121 genes were distributed among the 20 chromosomes and four scaffolds. Gene distribution was uneven between chromosomes. For example, 18 *PePUB* genes were located on chromosome 17, while only one *PePUB* gene was found on chromosome 19. Gene duplication also occurred, with three pairs of genes being duplicated in parallel. As shown in Supplementary Tables S3, S4; Figure 2C, 94 genes were derived from whole genome duplication (WGD) and segmental duplication; 20 genes were assigned to dispersed duplication blocks while four genes were assigned to tandem duplication blocks. Only 2.48% were derived from proximal duplication. These results suggest that WGD and segmental duplication events can cause the expansion of the *PUB* gene family. The relationship between *P. edulis*, *A. thaliana*, and rice is shown in Figure 2D. When compared with *PUB* genes in *P. edulis*, only 13 *PUB* homologs were found in the *A. thaliana* genome, while 93 *PUB* homologs were found in the rice genome. This shows that *P. edulis* and rice are genetically closely related. In addition, 13 *PUB* genes in rice had two homologous copies in *P. edulis*, while some had three copies. There were also 28 *PUB* genes in rice that had no corresponding copies in *P. edulis*. This may have resulted from a whole genome polyploidy event during the evolution of the *PUB* gene family.

### Cis-Regulatory Element Analyses of *PePUB* Genes

**FIGURE 3:**
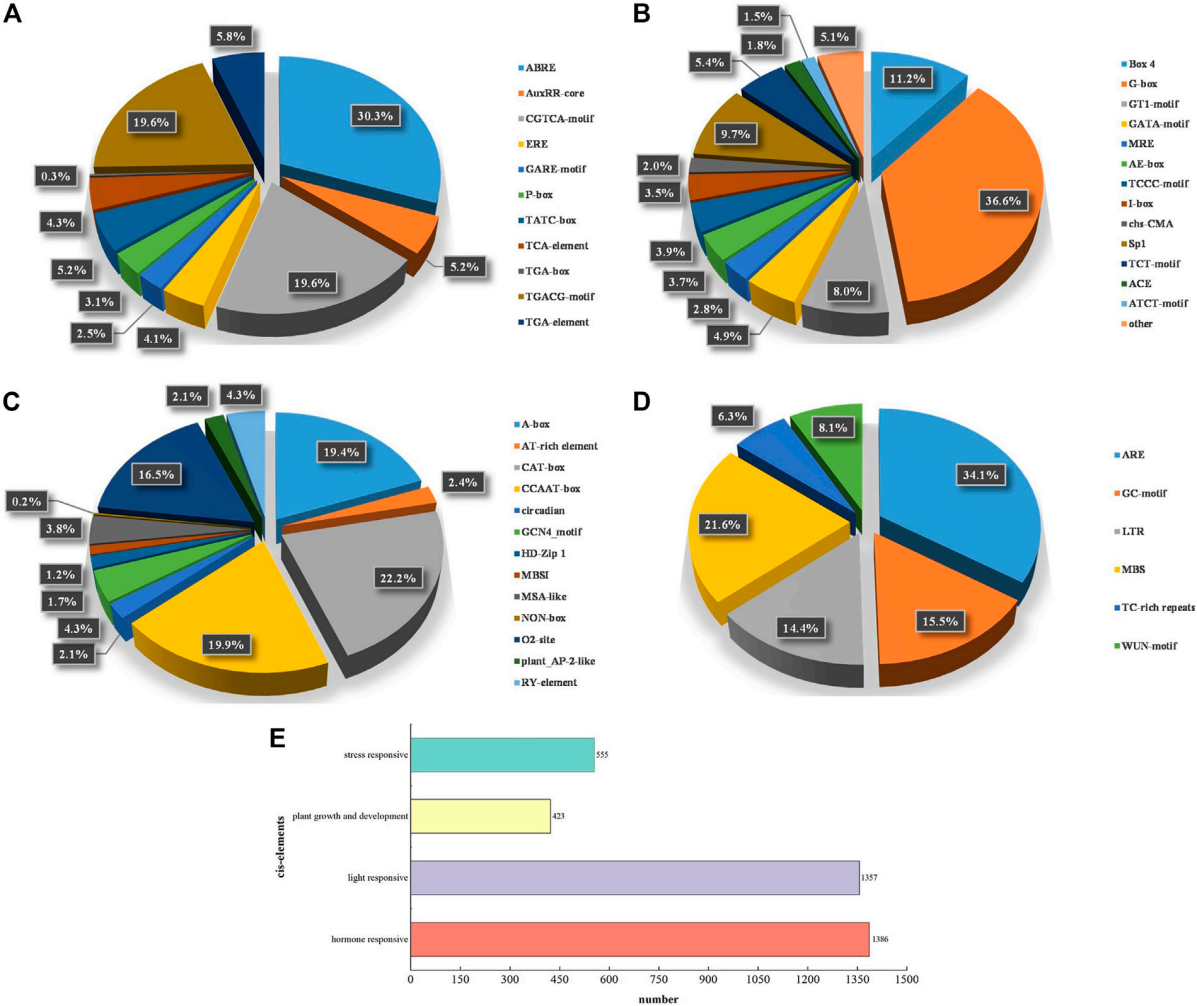
The analyses of cis-acting elements found in *PePUB* genes. **(A)** hormone-responsive, **(B)** light-responsive, **(C)** plant growth and development, **(D)** stress-responsive. **(E)** The sum of cis-elements in the four categories. Different colors indicate different cis-acting elements and the ratio of cis-elements present in *PePUB* genes.

We analyzed specific promoter regions of the 121 *PePUB* genes (Figure 3 and Supplementary Table S5) and examined the response of the cis-elements to biotic and abiotic stress. In addition to basic cis-elements, such as the CAAT-box and TATA-box, cis-elements are divided into four groups: hormone-responsive, light-responsive, plant growth and development-related, and stress-related. The results showed that 114 genes had hormone-responsive cis-elements, 117 genes had light-responsive cis-elements, 98 genes had plant growth and development-related cis-elements, and 116 genes had hormone-responsive cis-elements. Hormone-responsive cis-elements included ABRE, AuxRR-core, CGTCA-motif, ERE, GARE-motif, P-box, TATC-box, TCA-element, TGA-box, TGACG-motif, and TGA-element cis-elements. Light-responsive cis-elements included Box 4, G-box, GT1-motif, GATA-motif, MRE, AE-box, TCCC-motif, I-box, chs-CMA, Sp1, TCT-motif, ACE, and ATCT-motif. Development-related cis-elements included A-box, AT-box, AT-box, TCCC-motif, I-box, chs-CMA, Sp1, TCT-motif, ACE, and ATCT-motif rich element. Stress-related cis-elements included ARE, GC-motif, GCN4-motif, HD-Zip 1, MBSI, MSA-like, NON-box, O2-site, plant AP-2-like, and RY-element. LTR, MBS, TC-rich repeats, and WUN-motif are closely related to anaerobic induction, drought induction, low-temperature responsiveness, defense, stress responsiveness, wound-responsive elements, and hypoxia-specific induction.

### Expression Analyses of *PePUB* Genes in Different Tissues of Moso Bamboo Based on RNA-Seq Dataset

**FIGURE 4:**
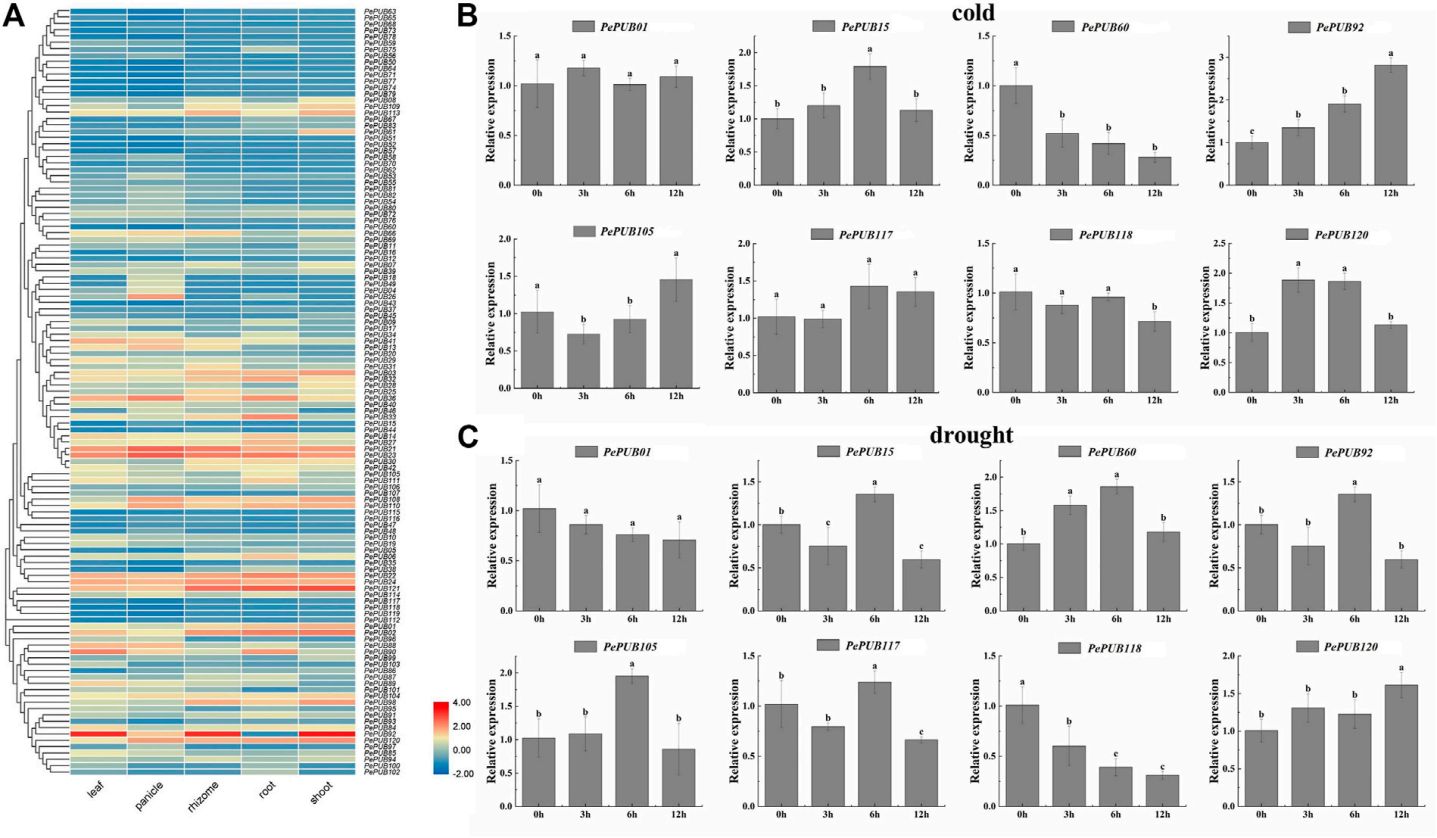
**(A)** Tissue-specific gene expression patterns of 121 *PePUB* genes. The expression patterns of 121 *PePUB* genes in leaf, panicle, rhizome, root, and shoot. The color scale illustrates the log2 (TPM + 1), red and blue colors reveal the high or low transcript abundance, respectively. **(B)** Expression profiles of eight candidate *PePUB* genes under low temperature treatment. **(C)** Expression profiles of eight candidate *PePUB* genes under drought stress. The values on X axis and Y axis represent the time point (hour) and the relative gene expression levels, respectively. The error strip represents three independent standard deviations (SD). Biological repetition. Different lowercase letters showed significant differences, which were determined by one-way analysis of variance, and then Duncan’s multi-range test (*p* < 0.05).

Expression heat maps of transcriptome data were created (Figure 4A) to explore the potential functions of the *PePUB* genes. We investigated the expression of *PePUB* genes in the leaves, panicles, rhizomes, roots, and shoots of *P. edulis*. We used fragments per kilobase of exon model per million mapped fragments (FPKM) to obtain *PePUB* gene expression abundance TPM and analyzed the expression profile of *PUB* gene by log2 (TPM + 1) values for the gene expression heat map. The results showed that the following fractions of TPM values were greater than 1: 72% (87/121) of the panicle, 65% (79/121) of the leaf, 55% (67/121) of the root, 51% (62/121) of the rhizome, and 43% (53/121) of the shoots.

### qRT-PCR Verification of Selected *PePUB* Genes in Different Stress

Low temperature and drought are the most important factors affecting the survival of *P. edulis*. Therefore, it is important to analyze whether *PePUB* genes are responsive to low-temperature and drought-stress treatments.

To test the role of *PePUB* genes under low temperature stress, eight *PePUB* genes from eight different subgroups (*PePUB01*, *PePUB15*, *PePUB60*, *PePUB92*, *PePUB105*, *PePUB117*, *PePUB118, and PePUB120*) were randomly selected for qRT-PCR analysis (Figure 4B). The expression levels of *PePUB15*, *PePUB92*, *PePUB118*, and *PePUB120* were upregulated at all treatment time points in low temperature conditions. However, *PePUB60* expression was downregulated throughout the treatment. The expression levels of both *PePUB92* and *PePUB105* were highest at 12 h *PePUB01* and *PePUB117* showed no significant difference in expression levels between the control and treatment groups.

To assess the response of *PePUB* genes to drought conditions, eight *PePUB* genes from different subgroups, including *PePUB01*, *PePUB15*, *PePUB60*, *PePUB92*, *PePUB105*, *PePUB117*, *PePUB118, and PePUB120*, were selected for qRT-PCR analysis (Figure 4C). The expression levels of three *PePUB* genes (*PePUB60*, *PePUB105*, *and PePUB117*) reached their highest levels at 6 h. Their expression patterns were different; *PePUB118* showed a decreasing pattern compared to that in the control group, and four *PePUB* genes (*PePUB01*, *PePUB15*, *PePUB92*, *and PePUB120*) did not significantly differ from those in the control group.

## Discussion

During growth and development, *P. edulis* is affected by various environmental and abiotic stresses, such as seasonal drought or flooding, and this can seriously affect the yield and quality of bamboo timber and bamboo shoots. Temperature and moisture are particularly important factors that limit the growth of *P. edulis*. In particular, soil salinization in China is another important limiting factor (Shi et al., 2020). The release of the first- and second-generation genome builds of *P. edulis* has significantly increased the identification of transcription factors and protein families involved in the regulation of *P. edulis* yield, quality, and stress tolerance (Zhang et al., 2005; Stone et al., 2006; Zhang et al., 2007). However, little is known about the *PUB* gene family in *P. edulis*. The *PUB* gene family has been shown to play important roles in several physiological activities in many plants, including phytohormones and light signaling. These genes are also involved in the regulation of cell cycle, transcription, and self-recognition in response to biotic and abiotic stresses. The properties and functions of the *PUB* gene family have been studied in a variety of plants. In this study, bioinformatics analysis of the *PUB* genes in the *P. edulis* genome identified 121 *PePUB* genes. These genes were found to be unevenly distributed among chromosomes. *PePUB* genes in the same subgroup were found to have similar gene structures, while gene structure differed among subgroups. The diversity of the gene structures further suggests that *PePUB* proteins may have diverse biological functions.

### The Function of Different Subgroups of *PePUB* Genes

The conserved U-box domain of the *PePUB* genes is often found in combination with other domains, such as ARM repeats, WD40 repeats, and TRR structures. The *PePUB* genes can be divided into eight subgroups based on phylogenetic analysis of the PUB domains. Group I in *P. edulis* consists of two genes, whereas in *A. thaliana*, there is only one gene (*AtUFD2*). *AtUFD2* contains a conserved domain similar to that of the yeast UFD2 protein and can interact with the AAA-type ATPase CDC48 to participate in the regulation of cell cycle, death, organelle formation, and other physiological activities. Group II contains the largest number of genes, with 47 genes in *P. edulis*, and 30 genes in *A. thaliana*. In addition to the U-box domain, Group II genes contain an ARM repeat at the C-terminus, which allows protein–protein interactions to regulate physiological activities in the cell. Group III contains 34 genes all of which have an isomerase domain. Group IV in *P. edulis* contains 21 genes, while in *A. thaliana*, there are eight genes. Group IV genes contain a kinase domain at the N-terminus of the U-box domain, indicating that these proteins are involved in signal transduction via phosphorylation, in addition to being modified by ubiquitination. Group V in *P. edulis* contains 12 genes, and in *A. Thaliana*, there are seven genes. Group V genes only have U-box domains, while WD40 repeats are involved in signaling, transcriptional regulation, and regulation of various cellular functions through protein interactions. Group VII in *P. edulis* consists of two genes. *PUBs* in group VII contain the TRR domain, a 34 aa protein repeating sequence that often exhibits scaffold-like structures and is involved in protein–protein interactions. Group VIII in *P. edulis* consists of two genes, while there are none in *A. thaliana*.

In the structural analysis of the *PePUB* genes, we systematically analyzed highly conserved sequences (as indicated by phylogenetic analysis) and found that PePUB proteins in the same subgroup share a remarkably similar sequence. We also found many cis-elements that were commonly associated with various developmental, adversity-response, and stress-response factors. Ubiquitin ligase regulates the ABA signaling pathway and ABA synthesis (Pankaj et al., 2021), resulting in the inhibition of leaf senescence (Lim et al., 2017). There were 103 *PePUB* genes that contained methyl jasmonate response (MeJA) cis-acting elements (TGACG and CGTCA). Both local and systemic responses are rapid and co-regulated by mechanical damage and herbivory. A previous study showed that ethylene and growth hormones can limit metabolism in tomato fruits through a light-signaling cascade (Aline et al., 2018). The CAT-box (GCCACT) and CGTCC were associated with meristematic expression and were found in 61 and 45 *PePUB* genes, respectively. TGA-elements (AACGAC) (Kumar et al., 2018) and the growth hormone suppressor core sequence (GGTCCAT) were found in 52 and 18 growth hormone-responsive *PePUB* genes, respectively. The HD-ZIP promoter found in seven *PePUB* genes provides protein-binding sites and is closely involved in leaf sarcomere differentiation and trichome development (Wang et al., 2013). Of the *PePUB* genes, 70 were found to contain the MBS motif (TAACTG), 32 genes were found with TC-rich repeats (GTTTTCTTAC), 61 genes with anaerobic induction-related cis-elements (GCCCCGG and TGGTTT), 91 genes with low-temperature and drought-response cis-regulation (GGCCGACAT), and 43 genes with other adversity-response elements. These elements were shown to rapidly regulate gene expression in response to changes in external stimuli. These findings provide a considerable advancement to our understanding of the molecular mechanisms of *PePUB* gene functions.

### Potential Functions and Expression Analyses of *PePUB* Genes

As previously mentioned, *P. edulis* is an important temperate woody bamboo species. Persistent and extremely low temperatures, annual effective cumulative temperature, and rainfall from March to April (the shooting period) and September (the rhizome growth period) are the main factors that limit the spread of the species (Shi et al., 2020). Previous studies have shown that *PUB* genes play an important role in the regulation of plant growth, development, and stress response (Azevedo et al., 2001). In this study, cis-element analysis of the *PePUB* promoters revealed the presence of elements associated with plant growth, development, and stress response. We also investigated the expression pattern of *PePUBs* in response to low temperatures and drought stress using qRT-PCR. Many studies have shown that *PUB* genes play an important role in the plant stress response. For example, overexpression of *AtCHIP* leads to increased sensitivity to temperature stress in *A. thaliana* (Yan et al., 2003). In this study, the *P. edulis* homolog, *PePUB120*, was identified with an identical protein class and subcellular localization. Significant gene expression changes in response to cold stress indicated that the homologous genes were functionally similar. We also found that the *PePUB15* and *PePUB92* genes responded positively to cold stress, and no homologs were found in *A. thaliana* or rice. qRT-PCR analysis showed high expression of *PePUB60* and *PePUB120* was in drought conditions. This suggests that *PePUB* genes play an important role in the response to adverse stress. In conclusion, we propose that *PePUB15*, *PePUB60*, *PePUB92*, and *PePUB120* are important candidates for improving *P. edulis* growth and quality.

## Conclusion

In this study, we investigated the *PUB* gene family in *P. edulis* by analyzing the genome and transcriptome, and identified 121 *PePUB* genes. To better understand the structure and function of *PePUB* genes, we analyzed their gene structure, protein motifs, and cis-acting elements in the promoter. WGD and segmental duplication were the main causes of the expansion of the *PUB* gene family. The expression pattern of *PePUB* genes was altered under different stress conditions. In addition, we found that *PePUB15*, *PePUB60*, *PePUB92*, and *PePUB120* responded positively to abiotic stress. This study provides a basis for a comprehensive understanding of the *PUB* genes in *P. edulis* and lays the foundation for future studies.

## Data Availability

The RNA-seq data were deposited in the Sequence Read Archive (SRA) of the National Center for Biotechnology Information (https://www.ncbi.nlm.nih.gov/sra) under the accession number SRX2408703.
